# Heatwaves and emergency department visits in the single hospital of arid Central Australia – a time series study

**DOI:** 10.1080/16549716.2026.2757371

**Published:** 2026-11-09

**Authors:** Mohammad Radwanur Talukder, Supriya Mathew, Rishu Thakur, Manoj Bhatta, Dung Phung, Syed Moshfiqur Rahman

**Affiliations:** a Women and Kids Theme, South Australian Health and Medical Research Institute, Adelaide, South Australia; b Global Health and Migration Unit, Department of Women’s and Children’s Health, Uppsala University, Uppsala, Sweden; c Menzies School of Health Research, Charles Darwin University, Alice Springs, NT, Australia; d School of Public Health, The University of Queensland, Brisbane, QLD, Australia; e Queensland Alliance for Environmental Health Sciences, The University of Queensland, Brisbane, QLD, Australia

**Keywords:** Remote, climate change, Central Australia, heatwave, temperature

## Abstract

**Background:**

The arid Central Australia will continue to experience increasing hotter temperatures.

**Objectives:**

This research assessed associations between heatwaves and all cause, and cause-specific emergency department (ED) visits for cardiovascular diseases, infectious diseases, kidney and urinary tract diseases (KD), mental disorders and respiratory diseases.

**Methods:**

Daily ED visits data, 2010 to 2021, from Alice Springs Hospital, Central Australia, were obtained. Nine heatwave definitions based on combinations of mean temperature thresholds (≥90th, ≥95th, and ≥97th percentile) and durations (≥2, ≥3, and ≥4 consecutive days) during warm months were assessed in quasi-Poisson regression models. The ≥ 95th percentile for ≥2 consecutive days was the primary exposure, with remaining definitions assessed in exploratory analyses.

**Results:**

A total of 50,324 ED visits were recorded. Heatwaves, ≥95th percentile of daily mean temperature for ≥2 days and ≥90th percentile temperature for ≥2 days, were associated with modest increases in all cause ED visits at lag 0 (HW2 relative risk – RR 1.02, 95% CI 0.97–1.07) compared to non-heatwave days. Significant increases in KD‑related ED visits were consistently observed for several heatwave thresholds including primary heatwaves exposure over several lag days (at lag 0-2 days cumulative RR 1.37, 95% CI 1.02–1.83). Increased risks of ED visits from heatwaves were also observed among people aged > 45 years.

**Conclusions:**

These findings suggest differential effects of heatwaves on ED visits in the single hospital in Central Australia and highlight need for emergency services preparedness within remote health service systems that is backed up by local demographic and health profiles.

## Paper Context

- **Main findings**: The study contributes to building evidence base on the health impacts of heatwaves in remote arid Central Australia.
- **Added knowledge**: Moderate to severe heatwaves are consistently associated with increased risks of kidney related emergency department visits. The effects of heatwaves on ED visits varied according to different heatwave intensity and duration.
- **Global health impact for policy and action**: This study supports the need for proactive emergency service response planning within the remote health service systems to reduce the health impacts from increasing extreme heat events.

## Background

Australia has reported an average warming of 1.5°C since the early 20^th^ century, with more frequent and extreme heat events resulting in significant health impacts from these exposures [1]. Extreme heat has resulted in more deaths and hospitalisations than any other extreme weather-related hazards in Australia [2]. From 2012–13 to 2021–22, nearly 80% of extreme weather-related hospitalisations (of a total 9,119) and from 2011–12 to 2020–21, over 40% of extreme weather-related deaths (of a total 677) were due to exposure to extreme heat [3].

Heat can cause a wide range of adverse and potentially fatal health outcomes, including heat exhaustion and heatstroke, and worsen underlying chronic illnesses such as cardiovascular disease (CVD), diabetes and asthma [4,5]. Young children, older adults, people with chronic conditions, and those living in socioeconomically disadvantaged or poorly ventilated environments are particularly at risk [6]. The most recent systematic review and meta-analysis including 16 observational studies among older adults (≥60 years) also showed significant increase in emergency department (ED) visits from heatwave exposure [7] Extreme hot temperatures negatively affect mental health and are associated with increased mortality and morbidity [4]. Ample research in Australia also reported increased risk of deaths from CVD, diabetes, respiratory disease and mental/behavioral disorders, and hospitalisations from renal disease, neurological disease, stroke, mental disorders, diabetes, and ischemic heart disease following extreme heat exposure [8]. Significant increases in health services demand ranging from hospital, emergency, and ambulance demand as well as deaths were observed across Australia during heatwave periods [9]. Despite having varying climate zones (i.e. tropical, desert and temperate regions) and remoteness structures (very remote inland communities with population sizes less than 500 separated by 1500 km from the nearest coastal urban centres) in Australia, heat health impact research has mostly focused on major coastal cities and tropical and temperate regions [8–10]. Recent research examining at statistical area level 2 (SA2) has indicated a wide regional variation in heat-related mortality across Australian communities [11]. Communities in the Northern Territory (NT), a jurisdiction with no urban centre exhibited attributable mortality rate (AMR) of 1.17 (95%CI: 0.60–1.62) deaths per 100,000 residents per year from heatwaves, the third highest across Australia. Within NT, Central Australian desert communities showed the highest AMR [11]. A recent study in Queensland also reported significant urban-rural disparities in the impact of heatwaves in ED visits [12]. An incremental increase in ED visits across urban, regional and rural Queensland was observed, ranging from a 6% increase in major cities to a 54% increase in very remote locations [12]. However, to date, limited research has assessed the impact of heatwaves on ED visits in Central Australia. A recent whole NT wide study reported significant increase in ED visits including in arid Central Australia from severe/ extreme heatwave intensity [13]. While ample research suggests that health effects of heatwaves can be delayed [8], study by [13] did not examine the lagged effects of heatwaves, which may have underestimated the association between heatwave and cause-specific ED visits. Information on both immediate and delayed effects of heat is essential for public health and health services preparedness.

Greatest increases in extreme temperatures are expected over northern Australia and in desert regions [14]. More frequent and longer spells of extreme temperatures as well as higher temperatures on the hottest days are projected in Central Australia [15]. For example, by 2050 temperatures are projected to rise by 2.6 ◦C than the 1986–2005 average and the annual average number of days over 35 ◦C is expected to nearly double in the Central Australia desert region [15]. Increases in extreme heat and heatwaves are more likely to increase heat-health burden among Central Australian remote populations that already experience a high disease burden compared to urban populations [16]. In the absence of universally accepted definition of a heatwave, studies have used different temperature thresholds and duration criteria to characterise extreme heat events [9]. Evaluation of multiple heatwave definitions allows a more comprehensive assessment of heat-related health risks and helps determine whether observed associations are robust to alternative characterisations of extreme heat. In remote Central Australia where exposure to extreme heat is frequent [15] and adaptive capacity, particularly among First Nations communities, may be compromised by housing quality, overcrowding, limited access to cooling infrastructure, and a higher burden of chronic health conditions [16,17]. Therefore, assessing health impacts across different heatwave thresholds can help identify which heatwave characteristics are most relevant for remote populations and improve the evidence base for targeted public health adaptation strategies. Understanding how heatwaves affect ED visits in Central Australia is important as it will help the single remote hospital’s ED surge capacity planning. In response to the increasing temperature concerns and potential demand on already strained ED, this study examined the effects of heatwaves on ED visits in arid Central Australia.

## Methods

### Study settings and data collection

#### Study area

Central Australia, with an area of approximately 600,000 square km, is a large outback region of NT, Australia [18] (a study area map was published previously [19]). Seventy-two per cent of approximately 43,000 population lives in Alice Springs, the major town in the region [18]. The region also has a high proportion of First Nations people [20], who also experience significant health issues. With an arid climate, Central Australia is largely represented by varying degrees of hot and dry weather with short and nocturnally cold winters [17]. The region’s summer average temperature ranges from 20 to 35°C, which can rise above 40°C. Winter temperature ranges from 3 to 20°C but can drop below 0°C [21]. Alice Springs Hospital (ASH), the 186-bed only specialised referral facility in the region, provides a range of acute and inpatient general medicine services.

#### Emergency department visits data

Daily counts of ED visits for all cause, cardiovascular disease (CVD), infectious diseases (ID), mental disorders (MD), kidney and urinary tract diseases (KD) and respiratory diseases (RD) at Alice Springs Hospital from January 2010 to 31 December 2021, were obtained from the Central Australia Health Data Registry. The selection of cause-specific disease conditions was based on most observed heat-health effects in the studies [8,9]. Disease classification was based on principal discharge diagnosis according to the International Classification of Diseases (ICD) 10 codes (Supplementary Table 1) [19,22]. Total all cause ED visits were also categorised by age (<18 years,18–45 years and above 45 years) considering the low life expectancy of First Nations people (65.6 years for males and 69.4 years for females) in the NT [23], sex (male and female), and First Nations status. Approval of the Central Australia Human Research Ethics Committee (NTHREC 21–4049) was obtained before commencing the data collection for this study.

#### Meteorological data

Daily minimum and maximum temperature, minimum and maximum relative humidity (RH) data were obtained for the Alice Springs Airport weather station from the Australian Scientific Information for Land Owners (SILO) dataset (https://www.longpaddock.qld.gov.au/silo/). SILO derives a spatially and temporally complete meteorological dataset by interpolating the raw data from the Bureau of Meteorology (BoM) observations across Australia [24]. Daily mean temperature was calculated from the average of daily minimum and maximum temperature.

### Statistical analysis

#### Definition of heatwaves

There is no consensus definition of heatwave. Heatwave is usually defined as a temperature above certain thresholds (ranging from 90^th^ to 99^th^ percentile) for a few consecutive days [25]. In this research, heatwaves were defined when daily mean temperatures were above a heat threshold (i.e. 90^th^, 95^th^ and 97^th^ percentiles of the daily mean temperature data) for ≥2, 3 or 4 consecutive days during the warm seasons (1 September to the end of May of next year) from 2010–2021 [21], resulting in 9 different definitions of heatwaves. In the main analyses, we presented heatwaves as the mean temperature above the 95^th^ percentile for ≥2 days during the warm months, 2010–2021, and for preceding 7 days. Based on previous research [26,27], the effects of multiple definitions of heatwaves were also explored to assess whether heatwave characteristics (intensity and duration) affect the risk on ED visits.

#### Impacts of heatwaves

We applied quasi-Poisson generalised linear regression models that account for overdispersion to assess the effects of heatwaves on ED visits for all cause and CVD, ID, KD, RD, and MD. Possible lagged effect of heatwaves on ED visits was examined using a distributed lag nonlinear model (DLNM) [27,28]. In DLNM, cross-basis (cb) matrix for each heatwave definition was created with a 7-day lag structure, a linear function and a natural cubic spline function of three degrees of freedom (dfs) to estimate the lagged effects of heatwaves. DLNMs also account for multicollinearity between lagged exposures and help prevent overestimating the immediate exposure effects [28].

The final built model was as follows:

$$\begin{array}{rl} & \mathrm{Yit}=\;\alpha +\mathrm{cb}\left(\mathrm{HWt},\mathrm{lag}=7\right)+\mathrm{ns}\left(\mathrm{Time},10\mathrm{df}/\mathrm{year}\right)\\ & \;\;\;\;\;\;\;\;\;\;\;\;+\mathrm{ns}\left(\mathrm{humidity},\mathrm{df}=3\right)+\mathrm{DOW}\\ & \;\;\;\;\;\;\;\;\;\;\;\;+\mathrm{holiday}+\mathrm{COVID} \end{array}$$

In this model, Yit refers to the estimated daily emergency presentations for disease i on day t; α represents the intercept; cb is a cross-basis function of heatwave on day t (HWt), lag is the number of lag days, and a 7-day lag structure is used to capture the single- and cumulative-lag effects of heatwaves based on previous studies [8,25]; ns is the natural cubic spline; natural cubic spline function of time (Time) with 10 degrees of freedom (df) per year controls long term trend and seasonality; Time is a variable that takes consecutive numbers (1,2,3 --, 3279) for the observation period (2010–2021) warm months. The optimal df for exposure lags and Time was determined using modified Akaike Information Criterion, the QAIC [29]. To control for potential confounding, the model was also adjusted for ns of mean RH (humidity) with 3 df, DOW (day of the week) is a categorical variable, holiday (Holiday) represents a binary variable for public holidays and COVID-19 (COVID) years (before 2020 and during or after 2020) as a binary variable. Cumulative lagged effect of heatwaves was considered based on previous research [26].

We also assessed the association between different heatwave definitions and all cause ED visits by sex (male, female), First Nations status and patient age (<18 years, 18–45 years, ≥45 years).

In sensitivity analyses we changed dfs for the natural cubic spline of cross-basis function (df = 4, 5), dfs for the natural cubic spline to control for seasonality and long-term trend (df = 8, 9), relative humidity (df = 4–5), extended lag effect up to 10 days, restricted to pre-COVID ED visits data, and excluded humidity in the model. We also applied excess heat factor (EHF), which is the Australian BoM’s benchmark for the heatwaves, using full ranges of EHF value and assess specific EHF percentiles (85^th^, 90^th^, and 95^th^) of EHF > 0 over lag days to measure the effects on ED visits. EHF was calculated in accordance with Nairn and Fawcett [30] and as described by BoM (pages 16–17) [31].

## Results

### Descriptive statistics

Of the total 50,324 ED visits at ASH during the warm months from 2010 to 2021, 55.9% were by females. Of the total, 37.9% (*n* = 19,084) were aged below 18 years, 39.3% (*n* = 19,793) were aged 18–45 years and 22.7% were above 45 years (*n* = 11,447). First Nations Australians contributed to 53% (*n* = 26,819) of the total ED visits. In terms of cause-specific ED visits, 40.6% of the total were RD related (*n* = 20,444), 26.6% were ID related (*n* = 13,386), 7.3% were KD related (*n* = 3686), and 5.7% were related to MD (*n* = 2889).

There was an average of 15.3 total ED visits (standard deviation of 5.5) per day, with a maximum of 50 total visits in a day. The average of total ED visits per day was higher among females than males, First Nations than non-Indigenous and for children < 18 years and 18–45 years than for those aged > 45 years (Table 1).

**Table 1.** Descriptive statistics of ED visits and meteorological variables, 2010–2021, in Central Australia.

Variable	Mean ± SD	Min	P25	P50	P75	P90	P95	Max
**Temperature**								
Mean	24.2±5.9	7.4	20.1	24.9	28.7	31.3	32.7	37.1
Minimum	16.1±6.4	−2.2	11.6	16.9	21.2	23.9	25.6	31
Maximum	32.3±6.3	11.6	28.1	33.2	37.2	39.8	41.2	45.7
**ED visits**								
All cause	15.3±5.5	2	11	15	18	23	25	50
Infectious	4±2.6	0	2	4	5	7	9	29
CVD^a^	0.7±0.8	0	0	0	1	2	2	5
Respiratory	6.3±3.4	1	4	6	8	11	12	25
KD^b^	1.1±1.1	0	0	1	2	3	3	7
MD^c^	0.9±0.9	0	0	1	1	2	3	5
**Demographic characteristics**								
Male	6.7±3.1	0	4	6	9	11	12	26
Female	8.6±3.6	1	6	8	11	13	15	31
First Nations	8.2±3.6	0	6	8	10	13	14	37
Non- Indigenous	7.1±3.5	0	5	7	9	12	14	30
Age groups (years)								
<18	5.8±3.1	0	4	5	8	10	11	32
18–45	6.0±2.8	0	4	6	8	10	11	22
>45	3.5±2.1	0	2	3	5	6	7	14

Note: a CVD – Cardiovascular disease.

b KD – Kidney and urinary tract diseases.

c MD – Mental disorders.

In total, there were 189 heatwave days with a threshold of ≥90^th^ percentile mean temperature for ≥2 days, i.e. an average of 21 heatwave days and 252 non-heatwave days per year during warm months (Table 2). The number of heatwave days was reduced with increased intensity and duration of the threshold temperature. For example, with a threshold of ≥97^th^ percentile mean temperature for ≥4 days, there were a total of 22 heatwave days for the period (Table 2).

**Table 2.** Heatwave definitions used in the analysis.

Heatwave (HW) definition	Threshold (percentile)	Mean temperature threshold value (°C)	Duration (days)	Total days
**Primary exposure**				
HW2	≥95^th^	32.8	≥2	91
**Secondary exposures**				
HW1	≥90^th^	31.4	≥2	189
HW3	≥97^th^	33.5	≥2	53
HW4	≥90^th^	31.4	≥3	121
HW5	≥95^th^	32.8	≥3	58
HW6	≥97^th^	33.5	≥3	34
HW7	≥90^th^	31.4	≥4	81
HW8	≥95^th^	32.8	≥4	40
HW9 (most severe heatwave)	≥97^th^	33.5	≥4	22

## Effect of heatwaves on ED visits

Figure 1 presents the effect of primary heatwaves, i.e. ≥95^th^ percentile temperature for 2 days or more (HW2), on ED visits over lag 0–7 days for all causes and cause-specific conditions in Central Australia. Primary heatwaves (HW2) were significantly associated with increased risks of KD‑related ED visits from lag 0 to lag 0–3 days (for example, at lag 0–2 days cumulative RR 1.37, 95% CI 1.02–1.1.83) (Figure 1). Significant increases in cumulative risks of KD‑related ED visits were also consistently observed for several heatwave thresholds (HW3, HW5, HW6, HW8, HW9) over several lag days (Figure 2, Supplementary Figures 1b, 1d, 1e, 1 g, 1 h Supplementary Table 2). For example, at lag 0–2 days, HW3, HW5 (consistent with Australian Bureau of Meteorology definition of heatwaves), HW6, HW8, HW9 significantly increased risks of KD‑related ED visits (Figure 2).

**Figure 1.**
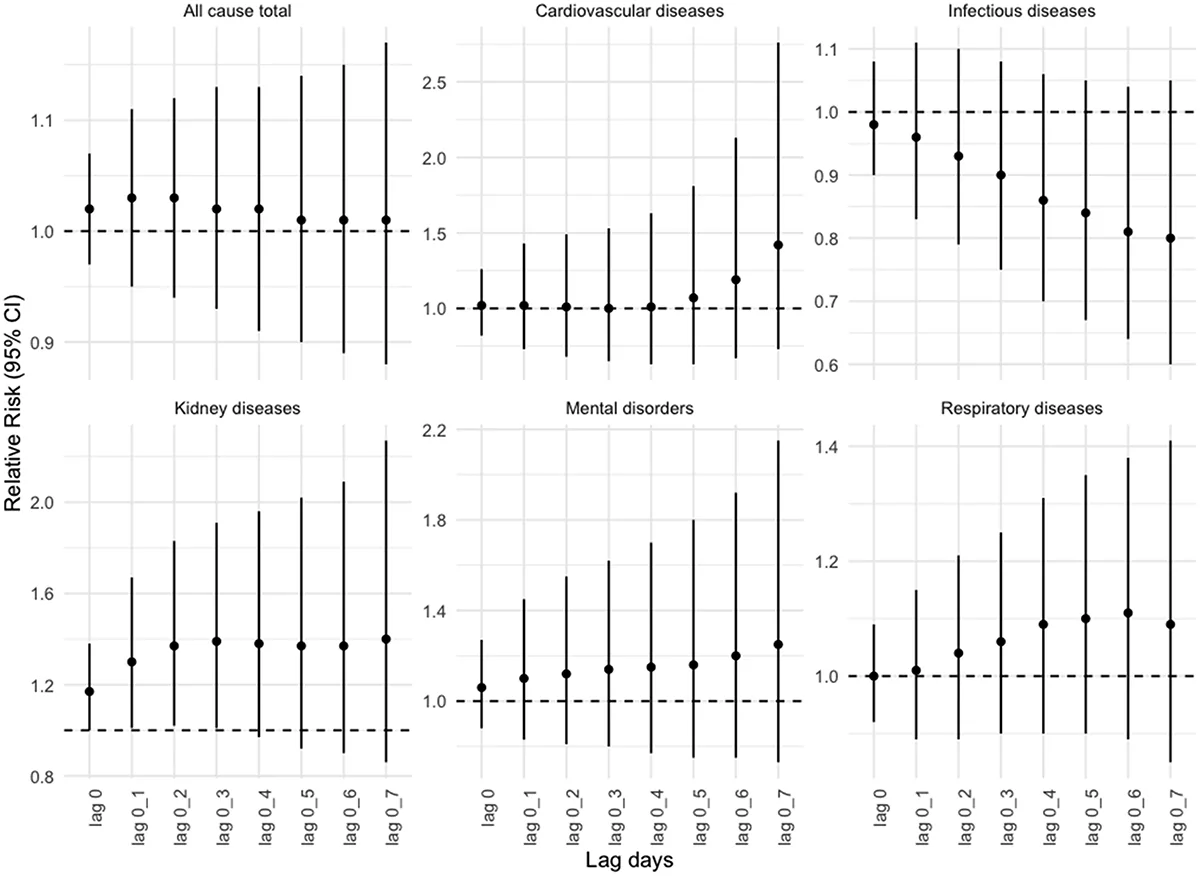
The cumulative relative risk of ED visits associated with primary heatwave expousre over lag 0-7 days.

**Figure 2.**
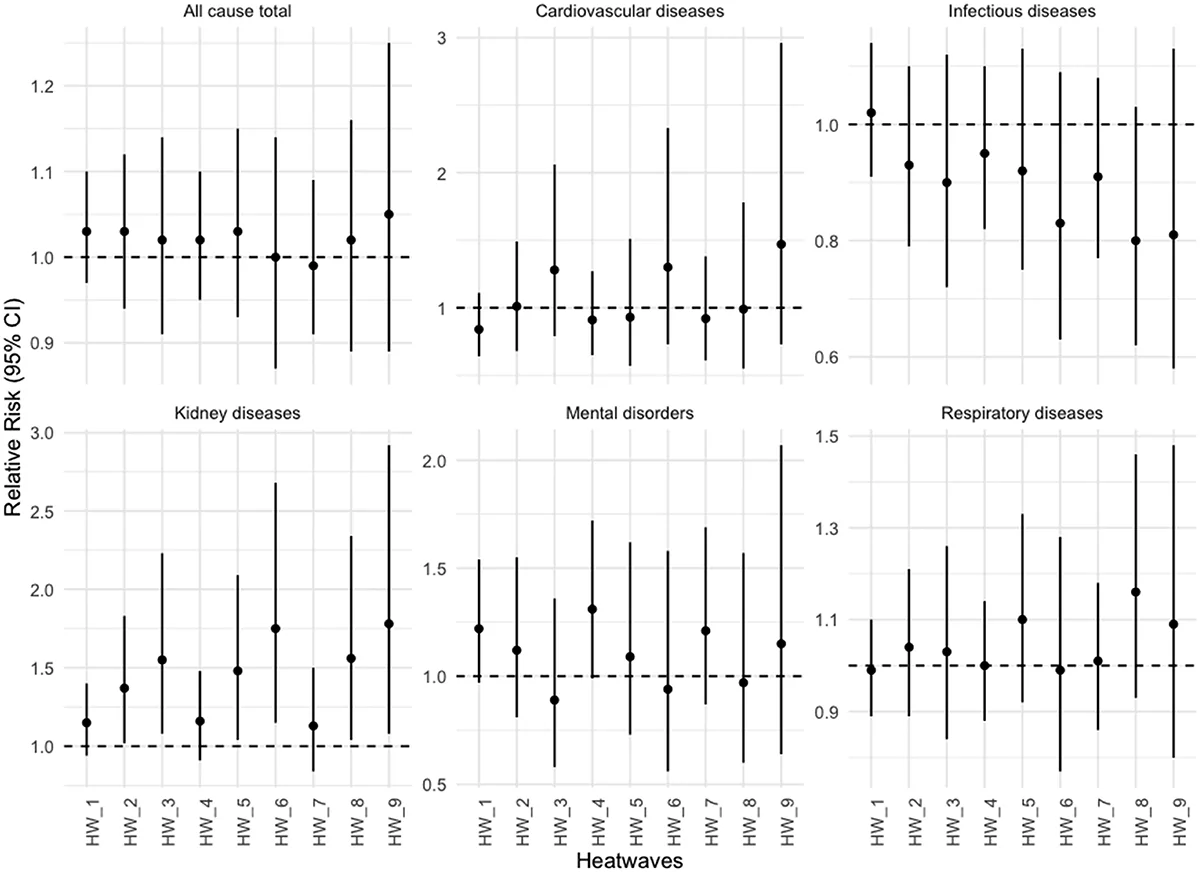
The cumulative effect of different heatwaves on ED visits over lag 0–2 days.

No significant effects of primary heatwaves defined as ≥95^th^ percentile mean temperature for ≥2 days were observed on all causes and other cause-specific conditions in this research. However, the effect of heatwaves on ED visits varied depending on different heatwave thresholds (Figure 2). Heatwaves, i.e. ≥95^th^ percentile mean temperature for ≥2 days (primary heatwaves-HW2) or ≥90^th^ percentile mean temperature for 2 days or more (HW1), were associated with modest increases in ED visits for all causes on same day (HW1 relative risk – RR 1.02, 95% CI 0.99–1.05; HW2 RR 1.02, 95% CI 0.97–1.07) compared to non-heatwave days suggesting possible acute effect (Supplementary Figure 1a, Supplementary Table 2). Significant increases in MD‑related ED visits were observed from lag 0–3 days to lag 0–5 days for HW1 and from lag 0–3 days to lag 0–4 days for HW4 (Supplementary Figure 1a,c, Supplementary Table 2). Increased risks of ED visits for respiratory diseases at lag 0–5 days were observed for HW8 and HW9 (Supplementary Figures 1 g, 1 h).

Examining the association between primary heatwaves and all cause ED visits by demographic characteristics an incremental increased risk of ED visits for people aged > 45 years was observed over lag 0–7 days with significant increased risks at lag 0–6 and lag 0–7 days (Figure 3) (Supplementary Table 3). For example, at lag 0-6 primary heatwaves increased risk (cumulative RR 1.29, 95% CI 1.00–1.65) of all cause ED visits for people aged > 45 years (Figure 3, Supplementary Table 3). Increased risks of ED visits for people aged > 45 years were also observed for HW3 at lag 0–7 days, for HW4 at lag 0–5 and lag 0–6 days and for HW7 at lag 0–5 days (Supplementary Table 3).

**Figure 3.**
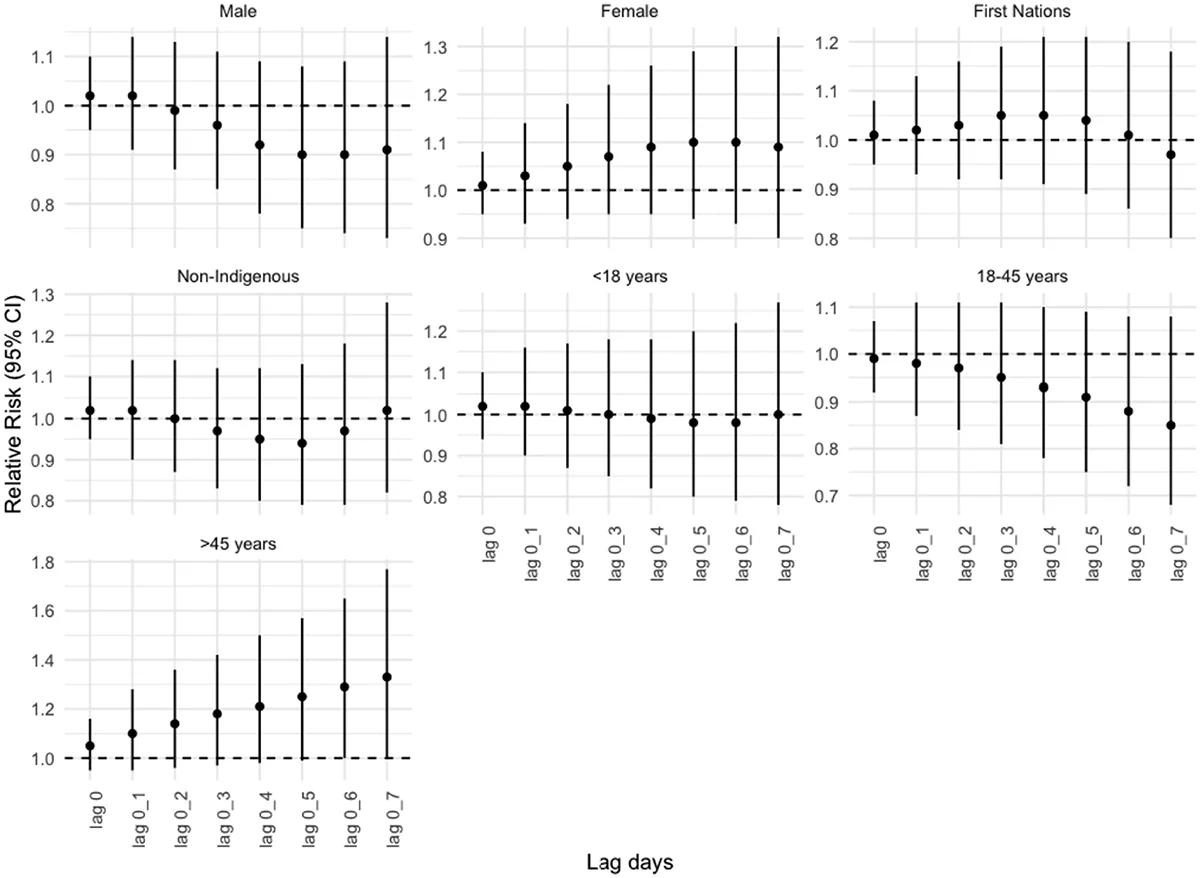
The cumulative relative risk of ED visits associated with primary heatwave exposure by demographic characteristics over lag 0-7 days.

In sensitivity analysis, the effects of heatwaves on all cause ED visits remained stable after changing the dfs (4, 5) for cross-basis function, dfs (4, 5) of natural cubic spline for relative humidity, dfs (8, 9) of natural cubic spline for seasonality and long-term trend and excluding humidity in the model (Supplementary Table 4). However, changing the dfs (df = 4) for the cross-basis function further strengthened the association between HW1 and all cause ED visits at lag 0 (RR 1.06, 95% CI 1.01–1.10) and lag 0–1 days. Similar changes in point estimates of RR also observed for primary heatwaves for same lag days. Extending the lagged effect up to 10 days also did not change the effect estimates (Supplementary Figure 4). Restricting the ED visits data to pre-COVID years, we have observed very similar point estimates in KD‑related ED visits to that obtained from the full study period (for example, at lag 0–2 days cumulative RR 1.35 vs RR 1.37 for the full study period) (Supplementary Figure 3). The loss of statistical significance as reflected in wider confidence intervals in the absence of a substantive change in effect size, suggests reduced precision due to the shorter observation period and smaller number of heatwave events.

In the sensitivity analysis of association between EHF and ED visits, using full ranges of EHF value (Supplementary Figure 2a) and specific EHF percentiles (85^th^, 90^th^, and 95^th^) of EHF > 0, showed no significant effect of heatwaves on all cause and cause-specific ED visits over lag days (Supplementary Figures S2b-2d)). Increasing trends of risks were observed for both KD and MD‑related ED visits for EHF ranges > 0 but they were not significant (Supplementary Figure 3a). Modest risks of KD‑related ED visits were observed for EHF percentiles (85^th^, 90^th^, and 95^th^) at lag 0–2 days (Supplementary Figure 2c).

## Discussion

Our research is one of the very few from arid Central Australia assessing the effects of heatwaves on ED visits and has shown that the effects of heatwaves on ED visits in Central Australia varied based on different heatwave intensity, duration and local acclimatisation. This observation is also consistent with several previous research [8,32]. Increased risks of KD‑related ED visits were consistently observed across several heatwave thresholds with increased intensity and duration from same day to lag 0–3 days. We also found significant associations between lower intensity heatwaves (≥90^th^ percentile mean temperature for ≥2 or ≥3 days) and MD at lag 0–3 days, and higher intensity and longer duration heatwaves (≥95^th^ and ≥ 97^th^ percentile mean temperatures for ≥4 days) and RD‑related ED visits at lag 0–5 days. Given the projected increase in the frequency and intensity of extreme heat in Central Australia [14], these findings contribute to a growing evidence base on the health impacts of heatwaves in remote Australian communities and highlight the need for locally relevant adaptation strategies [33].

Research on temperature-health effects in arid or remote Australia is generally scarce [10]. We have applied several heatwave definitions using mean temperature, as suggested in previous research [27], along with a priori primary heatwave exposure measure to assess which intensity and duration of heatwave thresholds show greatest risks on ED visits in arid climate. Our observation of modest increases in all cause ED visits from mild to moderate intensity and shorter duration heatwaves (≥90^th^ and ≥ 95^th^ percentile thresholds for ≥2 days) than that of more intense or prolonged heatwaves may be because of more frequent heatwave days using those intensity and shorter duration definitions resulting in better effect estimates compared to other heatwave metrices. Consequently, fewer exposure days from more restrictive heatwave thresholds, resulting in wider confidence intervals and greater uncertainty around the estimated health effects. Therefore, variation in statistical precision, rather than differences in the underlying health impacts of heatwaves, may partly explain inconsistencies in effect estimates across heatwave thresholds. The greater risks on the same day also suggest possible acute effect of heatwaves. Effect on emergency visits was the greatest from lower intensity (90^th^ percentile) and two-day-duration heatwaves in a tropical city in QLD [34]. Mild and short-lasting heatwaves may trigger pre-existing health conditions, which may lead to early medical help seeking [34]. A study in China also observed higher risk of ED visits from temperature intensity at or above 90^th^ percentile and two-day-duration heatwaves [35]. However, our observation is in contrast to a recent study that showed an overall increase in ED visits in arid Central Australia from severe/ extreme heatwave intensity applying EHF [13]. In sensitivity analyses, applying both full range of EHF and EHF > 0 over lag days we did not observe significant effects of EHF on all cause ED visits.

The finding that EHF-based heatwaves produced non-significant effects warrants further consideration. EHF is designed to capture both unusually hot conditions relative to long-term climate and short-term acclimatisation; however, its health relevance may vary across climatic regions. In addition to statistical imprecision described above, the absence of significant associations between heatwaves including EHF and ED visits may also reflect behavioural and physiological adaptation among population in regions that frequently experience high temperatures, thereby reducing adverse health impacts during heatwave periods. In chronically hot environments, EHF may identify heatwave episodes that are meteorologically extreme but do not necessarily exceed the adaptive capacity of residents [30]. Furthermore, ED visits represent only the more severe end of the health spectrum, and milder heat-related illnesses may be managed in primary health care services or through self-care without presenting to hospital emergency departments. Further research is therefore recommended to explore people’s behaviour and adaptation to extreme temperature and to examine any differential in health service uses between primary and tertiary health care services in Central Australia. A forthcoming paper from the research team is examining the association between temperature and primary health care uses in Central Australia (under review).

Like previous research [8,36], we observed a positive association between heatwaves and kidney and urinary tract diseases (KD)–related ED visits. Previous research in Central Australia showed elevated temperature was associated with increased risk of kidney‑related hospitalisations [19]. Collectively, these findings suggest that KD are one of the most heat-sensitive health outcomes in Central Australia. A QLD study observed a substantial increase in ED visits related to diseases of the genitourinary system (RR: 1.05, 95% CI: 1.00–1.09) during heatwave days [37]. A recent meta-analysis showed an increased risk of kidney disease morbidity from heatwave (pooled RR 1.059 (95% CI: 1.044–1.073) and increased pooled risk effects from medium (94^th^ to 96^th^ percentile temperature threshold) and high (97^th^ to 99^th^ percentile temperature threshold) intensity heatwaves than low intensity (90^th^ to 93^rd^ percentile temperature threshold) heatwaves. We also observed significant effects of heatwaves at or above the 95^th^ and 97^th^ percentiles of temperature on KD‑related ED visits [36]. Extreme temperatures can contribute to the development and exacerbation of urinary diseases in several ways. Increased core temperatures, dehydration, hypovolemia, blood hyperosmolality, electrolyte imbalance and hormonal (such as aldosterone and angiotensin) response from exposure to extreme heat affect kidney prefusion resulting in acute tubular damage and exacerbate kidney burden, therefore may predispose to AKI [36]. Excessive heat-induced sweating and dehydration can lead to increased concentration of calcium and uric acid, which in turn can contribute to the increased development of calculi [36]. Decreased urinary volume and flow partially affecting the eradication of pathogens, therefore, can exacerbate localised infections to sepsis and shock contributing to kidney damage [36,38]. Given the high burden of kidney disease in the NT and Central Australia [39], these findings highlight the importance of targeted heat-health interventions among community, patients with kidney diseases and health care providers to reduce the risk of heat-related deterioration in kidney function during heatwave periods.

We have also observed an increase in ED visits for MD from mild‑intensity heatwaves. While statistical imprecision stated above may partly explain these inconsistencies in effect estimates between heatwave thresholds, it is noteworthy that mental disorders related to substance use disorders, dementia and organic mental disorders were not considered in this study, which may underestimate the effect of heatwaves on mental disorders. To date, little is known about effects of extreme temperatures on MDs in arid Central Australia. Elsewhere in warm temperate Australia, heatwave showed an increase in MD‑related ED visits [8]. There is a lack of substantial data on the underlying mechanisms of mental health effects from extreme heat [40]. Extreme heat may affect mental health through several biological, psychological, and behavioural pathways. Heat exposure contributes to anxiety through the activation of the hypothalamic-pituitary-adrenal axis and the presence of inflammation and oxidative stress as the primary pathways. Higher levels of psychological stress could lead to the subsequent onset of mental illness. Evidence also indicates that poor physical health affects mental health [41]. The study also reported increased alcohol consumption as a potential coping mechanism from heat. It was hypothesised that higher levels of psychological stress or alcohol consumption would increase the risk of developing mental illness over the long term. Temperature-related sleep disruption also could contribute to the observed changes in mental health [40].

Unlike previous studies that have primarily examined the health effects of temperature including heatwaves among older adults of more advanced age (≥60 years) [7], our study identified increased risks in a relatively younger older-adult group (>45 years) in Central Australia. Thus, the health profiles of people in this age range, particularly of First Nations people in Central Australia, characterised by an earlier onset of chronic disease and a high burden of multimorbidity [16] may be comparable to those of older populations elsewhere, potentially compromising their thermoregulatory capacity during heat stress. Therefore, the finding suggests that age-based classifications of heat vulnerability should be tailored to local population contexts rather than relying solely on conventional age thresholds.

With the projected climate change, the Central Australia will experience more days of extreme temperatures. By 2050, temperature will be above 35°C for almost half of the year in Central Australia [15]. Even modest increases in ED demand therefore may place considerable pressure on its remote healthcare systems. Remote hospitals often operate with limited workforce capacity, restricted specialist services, and fewer surge resources [9]. As a result, relatively small increases in patient presentations during heatwave events may have disproportionate implications for service delivery. Heatwaves can also affect healthcare infrastructure through increased energy demand and transportation disruptions [9]. Therefore, the absence of strong epidemiological associations should not diminish the importance of heatwave preparedness within remote health services. To enhance emergency responses during heatwave events, proactive planning is needed to ensure that emergency services are adequately stocked with necessary cooling supplies, such as drinking water, ice, water spray bottles, fans, and cooling blankets [9]. Additional resources needed for local services should also be based on the demographic and disease profiles of the population in the community [9]. Coping with extreme temperatures, particularly for First Nations people, in Central Australia is constrained by poor housing infrastructure, energy insecurity affecting indoor thermal comfort, and limited availability of shaded outdoor areas or heat shelters [33]. Therefore, reducing the heat-health effects in Central Australia will also require multisectoral engagement and investment beyond health sector including human services, infrastructure and transport. Heat vulnerability index (HVI) based on future temperature changes is also necessary to accurately forecast vulnerability to heatwaves for Central Australian communities in the coming decades. Future heat-health studies in Central Australia should also consider validating and refining locally relevant heatwave thresholds using spatial and local exposure data, while also considering population vulnerability and adaptive capacity, healthcare accessibility and socio-economic status to improve understanding of geographical variations in health risks.

This research has several limitations. A potential limitation of this study is that we used fixed location Alice Springs Airport temperature data for exposure assessment, and individual-level exposure data were not available in this study. This may create exposure misclassification, particularly for residents living in dispersed remote locations (around 30% of the total Central Australian population). Such misclassification is likely to be non-differential with respect to ED visits, therefore, the observed effects of heatwaves on ED visits may have been underestimated. Furthermore, residual confounding from unmeasured individual- and area-level characteristics, such as housing conditions, air-conditioning use, and socioeconomic factors, cannot be ruled out, and may have influenced the magnitude of the observed heatwave effects. Use of temperature data from a single site compared to use of average temperature or spatiotemporal data yielded similar results in previous research [42]. International Classification of Disease (ICD)–based disease classifications may introduce outcome misclassification. However, this outcome bias is likely to be random and may underestimate the heatwaves-related risk. The association between heatwave and injury-related ED visits could not be considered in this paper because of administrative restriction to accessing already extracted historical ED visit data. We also did not account for air pollution data in the model. Evidence suggests that adjustment for air pollution has minimal influence on estimates of heat-related morbidity [43,44]. Moreover, certain air pollutants, particularly higher fine particulate matter and ozone levels due to extreme temperature, associated increase in energy demand and sunlight, may serve as a mediator between heat exposure and health outcomes [32,44,45]. Therefore, the observed associations should be interpreted as the overall health effects of heatwave exposure that are potentially mediated through concurrent changes in air quality [32], such as due to bushfire smoke or dust. As multiple heatwave definitions, exposure lags, and health outcome aggregations were evaluated without applying a formal correction for multiple testing, some statistically significant results may reflect type I error rather than true associations. However, our analyses were followed an a priori primary heatwave exposure. Moreover, the direction and magnitude of the associations of some health outcomes (KD‑related ED visits) were broadly consistent across several heatwave definitions, suggesting that the findings are unlikely to be explained entirely by type I error.

## Conclusion

Our research demonstrates increased risk of ED visits, more consistently related to kidney and urinary tract diseases from heatwaves in the single hospital in remote Central Australia. With projected increase in the frequency and intensity of extreme heat, even modest increases in ED demand are likely to pose additional burden within the remote health service systems. Proactive emergency service response planning consistent with demographic and health profiles in the local context is needed to ensure adequate supply of resources to reduce heat-health risks. These findings also support the need for multisectoral engagement and context-specific heat-health adaptation strategies, including heat-health early warning communications and more investments to improve housing, energy security and access to cooling infrastructures to reduce the burden on the remote ED services. Future heat-health studies in Central Australia should also consider validating and refining locally relevant heatwave thresholds using spatial and local exposure data, air quality and factors associated with population vulnerability and adaptive capacity in the local context.

## Supplementary Material

Supplementary Tables.docx

STROBE checklist_HW_CA.docx

## Data Availability

This study used secondary hospitalization data from Clinical Analytics NT Health, including data on First Nations people. Researchers can access the data from the corresponding author on reasonable request with written approval from the Northern Territory Human Research Ethics Committee (contact via ethics@menzies.edu.au) and with the permission of the NT Health Research Governance Office (contact via nthealth.rgo@nt.gov.au).
