# Supplementary material for: Heatwaves and emergency department visits in the single hospital of arid Central Australia – a time series study
Source: Glob Health Action. 2026 Nov 9;19(1):2757371. doi: 10.1080/16549716.2026.2757371 (PMC13757921; doi:10.1080/16549716.2026.2757371)
Supplement: Supplementary file 2 [file ZGHA_A_2757371_SM7276.docx]

**Supplementary Table 1** ICD 10 codes for disease classification

| **Disease classification** | **ICD-10 codes** |
| --- | --- |
| Cardiovascular diseases | I10-99  excluding acute rheumatic fever, I00-02, and chronic rheumatic heart diseases, I05-09 |
| Certain infectious and parasitic diseases | A00-B99  excluding infections with predominantly sexual modes of transmission, A50-64, HIV, B20-24, helminthiases, B65-83, sequelae of infectious and parasitic diseases, B90-94 |
| Mental disorders | F20-F79 |
| Respiratory diseases | J00-99  excluding lung diseases due to external agents, J60-70 |
| Kidney and urinary tract diseases | Nephron/tubular disorders, N00– N16; kidney disease N17–N19; and disorders of kidney and ureter, N20–N29 were used for kidney related total hospitalisation visits. Specific kidney conditions were diagnosed using following ICD codes - N17: AKI, N00–N08: glomerular diseases, N10–N16: renal tubulo-interstitial diseases, N18: CKD, N17–N19: kidney failure, N20–N23: urolithiasis and N39 for UTI |

**Supplementary Figure 1 a-h**

**1 a Cumulative effect of heatwaves (≥90^th^ percentile of mean temperature for ≥2 days) on ED visits**

**1 b Cumulative effect of heatwaves (≥97^th^ percentile of mean temperature for ≥2 days) on ED visits**

**1 c Cumulative effect of heatwaves (≥90^th^ percentile of mean temperature for ≥3 days) on ED visits**

**1 d Cumulative effect of heatwaves (≥95^th^ percentile of mean temperature for ≥3 days) on ED visits**

**1 e Cumulative effect of heatwaves (≥97^th^ percentile of mean temperature for ≥3 days) on ED visits**

**1f Cumulative effect of heatwaves (≥90^th^ percentile of mean temperature for ≥4 days) on ED visits**

**1g Cumulative effect of heatwaves (≥95^th^ percentile of mean temperature for ≥4 days) on ED visits**

**1h Cumulative effect of heatwaves (≥97^th^ percentile of mean temperature for ≥4 days) on ED visits**

**Supplementary Figures 2 a-d**

**2 a Cumulative effect of heatwaves defined as excess heat factor (EHF) on ED visits using full range of EHF**

**2b Cumulative effect of heatwaves defined as excess heat factor (EHF) on all cause and CVD related ED visits for EHF percentiles (85^th^, 90^th^, 95^th^)**

**2c** **Cumulative effect of heatwaves defined as excess heat factor (EHF) on infectious and kidney diseases related ED visits for EHF percentiles (85^th^, 90^th^, 95^th^)**

**2d** **Cumulative effect of heatwaves defined as excess heat factor (EHF) on mental disorders and respiratory diseases related ED visits for EHF percentiles (85^th^, 90^th^, 95^th^)**

**Supplementary Figure 3 Cumulative effect of primary heatwaves (≥95^th^ percentile of mean temperature for ≥2 days) on ED visits only pre-COVID period**

**Supplementary Figure 4 Cumulative effect of primary heatwaves (≥95^th^ percentile of mean temperature for ≥2 days) on ED visits over lag 0-10 days**

**Supplementary Table 2** The cumulative relative risk of heatwaves on ED visits over lag 0-7 days

| **Lag days** | **Outcomes** | **HW1** | **95% CI** | **HW3** | **95% CI** | **HW4** | **95% CI** | **HW5** | **95% CI** | **HW6** | **95% CI** | **HW7** | **95% CI** | **HW8** | **95% CI** | **HW9** | **95% CI** |
| --- | --- | --- | --- | --- | --- | --- | --- | --- | --- | --- | --- | --- | --- | --- | --- | --- | --- |
| lag 0 | All cause total | 1.02 | 0.99, 1.05 | 1.01 | 0.94, 1.08 | 1.01 | 0.97, 1.05 | 1.02 | 0.96, 1.08 | 0.99 | 0.91, 1.07 | 1.00 | 0.95, 1.05 | 1.01 | 0.94, 1.09 | 1.02 | 0.92, 1.13 |
| lag 0_1 | All cause total | 1.03 | 0.98, 1.08 | 1.01 | 0.92, 1.12 | 1.02 | 0.95, 1.08 | 1.03 | 0.94, 1.13 | 0.99 | 0.87, 1.12 | 0.99 | 0.92, 1.08 | 1.02 | 0.90, 1.14 | 1.04 | 0.89, 1.21 |
| lag 0_2 | All cause total | 1.03 | 0.97, 1.10 | 1.02 | 0.91, 1.14 | 1.02 | 0.95, 1.10 | 1.03 | 0.93, 1.15 | 1.00 | 0.87, 1.14 | 0.99 | 0.91, 1.09 | 1.02 | 0.89, 1.16 | 1.05 | 0.89, 1.25 |
| lag 0_3 | All cause total | 1.03 | 0.96, 1.10 | 1.02 | 0.91, 1.16 | 1.02 | 0.94, 1.11 | 1.03 | 0.92, 1.16 | 1.01 | 0.87, 1.17 | 1.00 | 0.90, 1.10 | 1.02 | 0.89, 1.17 | 1.07 | 0.90, 1.28 |
| lag 0_4 | All cause total | 1.03 | 0.95, 1.11 | 1.03 | 0.90, 1.17 | 1.02 | 0.93, 1.11 | 1.02 | 0.90, 1.16 | 1.03 | 0.87, 1.21 | 1.00 | 0.90, 1.11 | 1.02 | 0.88, 1.18 | 1.09 | 0.90, 1.32 |
| lag 0_5 | All cause total | 1.02 | 0.94, 1.11 | 1.03 | 0.89, 1.19 | 1.01 | 0.92, 1.12 | 1.02 | 0.89, 1.17 | 1.04 | 0.87, 1.24 | 1.00 | 0.89, 1.12 | 1.02 | 0.87, 1.19 | 1.10 | 0.90, 1.35 |
| lag 0_6 | All cause total | 1.02 | 0.93, 1.12 | 1.03 | 0.88, 1.20 | 1.01 | 0.91, 1.12 | 1.01 | 0.87, 1.17 | 1.05 | 0.87, 1.26 | 1.01 | 0.89, 1.14 | 1.02 | 0.86, 1.20 | 1.11 | 0.90, 1.38 |
| lag 0_7 | All cause total | 1.02 | 0.92, 1.13 | 1.02 | 0.85, 1.22 | 1.01 | 0.89, 1.14 | 1.01 | 0.85, 1.19 | 1.05 | 0.85, 1.29 | 1.01 | 0.88, 1.16 | 1.02 | 0.85, 1.23 | 1.12 | 0.87, 1.43 |
| lag 0 | Cardiovascular diseases | 0.90 | 0.77, 1.04 | 1.12 | 0.85, 1.49 | 0.90 | 0.75, 1.08 | 0.92 | 0.70, 1.20 | 1.17 | 0.83, 1.64 | 0.88 | 0.70, 1.10 | 0.95 | 0.69, 1.33 | 1.38 | 0.90, 2.10 |
| lag 0_1 | Cardiovascular diseases | 0.85 | 0.67, 1.07 | 1.22 | 0.80, 1.86 | 0.87 | 0.66, 1.17 | 0.90 | 0.59, 1.37 | 1.27 | 0.76, 2.13 | 0.86 | 0.60, 1.21 | 0.95 | 0.57, 1.59 | 1.55 | 0.82, 2.91 |
| lag 0_2 | Cardiovascular diseases | 0.84 | 0.64, 1.11 | 1.28 | 0.79, 2.06 | 0.91 | 0.65, 1.27 | 0.93 | 0.57, 1.51 | 1.30 | 0.73, 2.33 | 0.92 | 0.61, 1.38 | 0.99 | 0.55, 1.78 | 1.47 | 0.73, 2.96 |
| lag 0_3 | Cardiovascular diseases | 0.87 | 0.64, 1.18 | 1.31 | 0.79, 2.19 | 0.98 | 0.68, 1.42 | 0.99 | 0.59, 1.68 | 1.29 | 0.69, 2.40 | 1.04 | 0.67, 1.61 | 1.06 | 0.56, 1.99 | 1.25 | 0.59, 2.67 |
| lag 0_4 | Cardiovascular diseases | 0.92 | 0.65, 1.29 | 1.33 | 0.75, 2.36 | 1.08 | 0.72, 1.63 | 1.07 | 0.60, 1.90 | 1.26 | 0.63, 2.52 | 1.19 | 0.74, 1.92 | 1.13 | 0.57, 2.26 | 1.08 | 0.46, 2.54 |
| lag 0_5 | Cardiovascular diseases | 0.97 | 0.67, 1.42 | 1.36 | 0.72, 2.55 | 1.18 | 0.75, 1.85 | 1.12 | 0.59, 2.09 | 1.24 | 0.58, 2.69 | 1.30 | 0.78, 2.18 | 1.19 | 0.57, 2.52 | 1.03 | 0.40, 2.68 |
| lag 0_6 | Cardiovascular diseases | 1.02 | 0.68, 1.54 | 1.40 | 0.71, 2.77 | 1.24 | 0.76, 2.02 | 1.12 | 0.57, 2.21 | 1.27 | 0.56, 2.91 | 1.31 | 0.76, 2.29 | 1.22 | 0.55, 2.69 | 1.17 | 0.42, 3.25 |
| lag 0_7 | Cardiovascular diseases | 1.06 | 0.66, 1.69 | 1.46 | 0.66, 3.23 | 1.26 | 0.72, 2.20 | 1.07 | 0.49, 2.33 | 1.36 | 0.52, 3.54 | 1.21 | 0.64, 2.29 | 1.20 | 0.49, 2.94 | 1.65 | 0.52, 5.20 |
| lag 0 | Respiratory diseases | 0.99 | 0.94, 1.05 | 0.99 | 0.88, 1.11 | 1.00 | 0.93, 1.07 | 1.03 | 0.93, 1.14 | 0.93 | 0.80, 1.08 | 1.00 | 0.92, 1.09 | 1.05 | 0.92, 1.19 | 0.96 | 0.80, 1.15 |
| lag 0_1 | Respiratory diseases | 0.99 | 0.90, 1.08 | 1.00 | 0.84, 1.19 | 1.00 | 0.90, 1.12 | 1.06 | 0.90, 1.25 | 0.93 | 0.75, 1.16 | 1.00 | 0.88, 1.15 | 1.10 | 0.90, 1.35 | 0.99 | 0.75, 1.31 |
| lag 0_2 | Respiratory diseases | 0.99 | 0.89, 1.10 | 1.03 | 0.84, 1.26 | 1.00 | 0.88, 1.14 | 1.10 | 0.92, 1.33 | 0.99 | 0.77, 1.28 | 1.01 | 0.86, 1.18 | 1.16 | 0.93, 1.46 | 1.09 | 0.80, 1.48 |
| lag 0_3 | Respiratory diseases | 0.99 | 0.88, 1.11 | 1.07 | 0.86, 1.33 | 1.00 | 0.86, 1.15 | 1.15 | 0.94, 1.40 | 1.09 | 0.83, 1.43 | 1.01 | 0.86, 1.20 | 1.23 | 0.97, 1.57 | 1.23 | 0.89, 1.70 |
| lag 0_4 | Respiratory diseases | 0.99 | 0.87, 1.13 | 1.11 | 0.87, 1.42 | 0.99 | 0.85, 1.16 | 1.19 | 0.95, 1.48 | 1.20 | 0.89, 1.61 | 1.02 | 0.85, 1.22 | 1.29 | 0.99, 1.67 | 1.38 | 0.97, 1.96 |
| lag 0_5 | Respiratory diseases | 0.99 | 0.85, 1.14 | 1.14 | 0.88, 1.49 | 0.99 | 0.83, 1.17 | 1.21 | 0.95, 1.54 | 1.26 | 0.92, 1.74 | 1.03 | 0.85, 1.26 | 1.32 | 1.00, 1.74 | 1.46 | 1.00, 2.13 |
| lag 0_6 | Respiratory diseases | 0.97 | 0.83, 1.13 | 1.15 | 0.87, 1.52 | 0.98 | 0.82, 1.18 | 1.21 | 0.94, 1.57 | 1.24 | 0.89, 1.74 | 1.05 | 0.85, 1.29 | 1.32 | 0.99, 1.77 | 1.43 | 0.96, 2.12 |
| lag 0_7 | Respiratory diseases | 0.94 | 0.78, 1.12 | 1.13 | 0.83, 1.55 | 0.98 | 0.79, 1.21 | 1.20 | 0.90, 1.60 | 1.13 | 0.77, 1.65 | 1.07 | 0.84, 1.35 | 1.29 | 0.93, 1.78 | 1.26 | 0.80, 2.00 |
| lag 0 | Infectious diseases | 1.02 | 0.96, 1.09 | 0.96 | 0.85, 1.09 | 0.98 | 0.91, 1.06 | 0.99 | 0.88, 1.11 | 0.93 | 0.79, 1.10 | 0.96 | 0.87, 1.06 | 0.89 | 0.77, 1.03 | 0.89 | 0.73, 1.08 |
| lag 0_1 | Infectious diseases | 1.03 | 0.93, 1.13 | 0.93 | 0.77, 1.13 | 0.97 | 0.86, 1.09 | 0.96 | 0.80, 1.15 | 0.88 | 0.69, 1.12 | 0.93 | 0.80, 1.08 | 0.83 | 0.66, 1.04 | 0.83 | 0.61, 1.12 |
| lag 0_2 | Infectious diseases | 1.02 | 0.91, 1.14 | 0.90 | 0.72, 1.12 | 0.95 | 0.82, 1.10 | 0.92 | 0.75, 1.13 | 0.83 | 0.63, 1.09 | 0.91 | 0.77, 1.08 | 0.80 | 0.62, 1.03 | 0.81 | 0.58, 1.13 |
| lag 0_3 | Infectious diseases | 1.00 | 0.88, 1.13 | 0.87 | 0.68, 1.10 | 0.93 | 0.80, 1.09 | 0.88 | 0.71, 1.10 | 0.80 | 0.60, 1.07 | 0.89 | 0.74, 1.08 | 0.80 | 0.61, 1.04 | 0.82 | 0.58, 1.16 |
| lag 0_4 | Infectious diseases | 0.97 | 0.84, 1.12 | 0.84 | 0.65, 1.09 | 0.92 | 0.77, 1.09 | 0.85 | 0.66, 1.08 | 0.78 | 0.57, 1.07 | 0.88 | 0.72, 1.08 | 0.81 | 0.61, 1.08 | 0.84 | 0.58, 1.21 |
| lag 0_5 | Infectious diseases | 0.95 | 0.81, 1.12 | 0.81 | 0.61, 1.08 | 0.90 | 0.74, 1.09 | 0.82 | 0.63, 1.07 | 0.78 | 0.55, 1.10 | 0.87 | 0.69, 1.08 | 0.81 | 0.60, 1.10 | 0.84 | 0.56, 1.25 |
| lag 0_6 | Infectious diseases | 0.94 | 0.79, 1.12 | 0.80 | 0.59, 1.07 | 0.88 | 0.71, 1.08 | 0.82 | 0.62, 1.08 | 0.80 | 0.56, 1.14 | 0.85 | 0.67, 1.07 | 0.81 | 0.59, 1.11 | 0.82 | 0.54, 1.25 |
| lag 0_7 | Infectious diseases | 0.93 | 0.76, 1.14 | 0.78 | 0.56, 1.09 | 0.86 | 0.67, 1.09 | 0.83 | 0.61, 1.13 | 0.84 | 0.56, 1.26 | 0.83 | 0.64, 1.09 | 0.79 | 0.55, 1.12 | 0.77 | 0.47, 1.26 |
| lag 0 | Kidney diseases | 1.06 | 0.95, 1.18 | 1.21 | 0.98, 1.50 | 1.07 | 0.93, 1.22 | 1.22 | 1.00, 1.49 | 1.35 | 1.04, 1.74 | 1.06 | 0.90, 1.25 | 1.31 | 1.03, 1.66 | 1.45 | 1.06, 1.97 |
| lag 0_1 | Kidney diseases | 1.11 | 0.93, 1.32 | 1.40 | 1.01, 1.93 | 1.12 | 0.91, 1.38 | 1.39 | 1.02, 1.88 | 1.62 | 1.10, 2.37 | 1.10 | 0.85, 1.42 | 1.51 | 1.05, 2.17 | 1.74 | 1.10, 2.74 |
| lag 0_2 | Kidney diseases | 1.15 | 0.94, 1.40 | 1.55 | 1.08, 2.23 | 1.16 | 0.91, 1.48 | 1.48 | 1.04, 2.09 | 1.75 | 1.15, 2.68 | 1.13 | 0.84, 1.50 | 1.56 | 1.04, 2.34 | 1.78 | 1.08, 2.92 |
| lag 0_3 | Kidney diseases | 1.17 | 0.94, 1.47 | 1.66 | 1.13, 2.44 | 1.18 | 0.90, 1.54 | 1.49 | 1.03, 2.16 | 1.76 | 1.13, 2.75 | 1.13 | 0.83, 1.54 | 1.49 | 0.97, 2.30 | 1.63 | 0.97, 2.74 |
| lag 0_4 | Kidney diseases | 1.19 | 0.92, 1.53 | 1.70 | 1.11, 2.61 | 1.18 | 0.88, 1.59 | 1.46 | 0.97, 2.20 | 1.69 | 1.03, 2.78 | 1.12 | 0.80, 1.57 | 1.38 | 0.86, 2.23 | 1.45 | 0.81, 2.58 |
| lag 0_5 | Kidney diseases | 1.20 | 0.91, 1.58 | 1.70 | 1.07, 2.70 | 1.17 | 0.85, 1.62 | 1.42 | 0.90, 2.22 | 1.62 | 0.94, 2.79 | 1.10 | 0.76, 1.59 | 1.31 | 0.78, 2.19 | 1.35 | 0.71, 2.56 |
| lag 0_6 | Kidney diseases | 1.20 | 0.89, 1.62 | 1.64 | 1.00, 2.69 | 1.14 | 0.80, 1.62 | 1.38 | 0.86, 2.23 | 1.57 | 0.88, 2.81 | 1.06 | 0.72, 1.58 | 1.29 | 0.75, 2.22 | 1.39 | 0.71, 2.75 |
| lag 0_7 | Kidney diseases | 1.20 | 0.85, 1.69 | 1.54 | 0.87, 2.73 | 1.10 | 0.73, 1.65 | 1.36 | 0.79, 2.36 | 1.57 | 0.80, 3.09 | 1.02 | 0.65, 1.60 | 1.36 | 0.74, 2.52 | 1.63 | 0.74, 3.58 |
| lag 0 | Mental disorders | 1.07 | 0.95, 1.21 | 0.92 | 0.72, 1.18 | 1.13 | 0.97, 1.31 | 1.04 | 0.83, 1.31 | 0.95 | 0.70, 1.30 | 1.10 | 0.91, 1.33 | 0.99 | 0.75, 1.32 | 1.06 | 0.74, 1.52 |
| lag 0_1 | Mental disorders | 1.15 | 0.95, 1.39 | 0.89 | 0.60, 1.30 | 1.23 | 0.97, 1.56 | 1.07 | 0.76, 1.52 | 0.93 | 0.58, 1.49 | 1.17 | 0.87, 1.57 | 0.98 | 0.64, 1.51 | 1.11 | 0.65, 1.90 |
| lag 0_2 | Mental disorders | 1.22 | 0.97, 1.54 | 0.89 | 0.58, 1.36 | 1.31 | 0.99, 1.72 | 1.09 | 0.73, 1.62 | 0.94 | 0.56, 1.58 | 1.21 | 0.87, 1.69 | 0.97 | 0.60, 1.57 | 1.15 | 0.64, 2.07 |
| lag 0_3 | Mental disorders | 1.30 | 1.01, 1.67 | 0.92 | 0.58, 1.45 | 1.36 | 1.00, 1.83 | 1.10 | 0.72, 1.68 | 0.98 | 0.58, 1.67 | 1.23 | 0.86, 1.77 | 0.96 | 0.58, 1.60 | 1.20 | 0.65, 2.18 |
| lag 0_4 | Mental disorders | 1.36 | 1.02, 1.80 | 0.98 | 0.60, 1.60 | 1.39 | 0.99, 1.95 | 1.12 | 0.70, 1.78 | 1.05 | 0.59, 1.86 | 1.26 | 0.85, 1.87 | 0.98 | 0.56, 1.69 | 1.26 | 0.66, 2.42 |
| lag 0_5 | Mental disorders | 1.39 | 1.01, 1.91 | 1.05 | 0.62, 1.79 | 1.43 | 0.98, 2.08 | 1.15 | 0.69, 1.90 | 1.16 | 0.63, 2.16 | 1.32 | 0.86, 2.03 | 1.02 | 0.57, 1.83 | 1.38 | 0.68, 2.79 |
| lag 0_6 | Mental disorders | 1.40 | 0.99, 1.97 | 1.15 | 0.65, 2.02 | 1.48 | 0.99, 2.23 | 1.20 | 0.70, 2.06 | 1.32 | 0.69, 2.55 | 1.42 | 0.90, 2.25 | 1.10 | 0.60, 2.03 | 1.57 | 0.75, 3.32 |
| lag 0_7 | Mental disorders | 1.37 | 0.92, 2.03 | 1.26 | 0.66, 2.41 | 1.56 | 0.97, 2.48 | 1.28 | 0.69, 2.38 | 1.56 | 0.73, 3.31 | 1.59 | 0.95, 2.69 | 1.24 | 0.62, 2.46 | 1.88 | 0.79, 4.48 |

**Supplementary Table 3** The cumulative relative risk of heatwaves on all cause ED visits by demographic characteristics over lag 0-7 days

|  |  | **HW1** | **HW3** | **HW4** | **HW5** | **HW6** | **HW7** | **HW8** | **HW9** |
| --- | --- | --- | --- | --- | --- | --- | --- | --- | --- |
| **Lag days** | **Characteristics** | **RR (95% CI)** | **RR (95% CI)** | **RR (95% CI)** | **RR (95% CI)** | **RR (95% CI)** | **RR (95% CI)** | **RR (95% CI)** | **RR (95% CI)** |
| lag 0 | Male | 1.03 (0.98–1.08) | 0.99 (0.90–1.10) | 1.02 (0.96–1.08) | 1.01 (0.92–1.11) | 0.95 (0.83–1.08) | 0.99 (0.92–1.08) | 1.00 (0.89–1.13) | 0.99 (0.84–1.16) |
| lag 0_1 | Male | 1.04 (0.96–1.12) | 0.98 (0.84–1.14) | 1.02 (0.92–1.12) | 1.00 (0.87–1.15) | 0.91 (0.75–1.11) | 0.98 (0.87–1.10) | 0.98 (0.82–1.17) | 0.97 (0.77–1.23) |
| lag 0_2 | Male | 1.03 (0.94–1.12) | 0.96 (0.81–1.14) | 1.00 (0.89–1.12) | 0.97 (0.82–1.14) | 0.89 (0.72–1.10) | 0.95 (0.83–1.09) | 0.95 (0.78–1.16) | 0.95 (0.73–1.23) |
| lag 0_3 | Male | 1.01 (0.91–1.11) | 0.94 (0.78–1.13) | 0.98 (0.86–1.11) | 0.93 (0.78–1.12) | 0.88 (0.70–1.10) | 0.93 (0.80–1.08) | 0.91 (0.73–1.12) | 0.92 (0.71–1.21) |
| lag 0_4 | Male | 0.99 (0.88–1.11) | 0.91 (0.74–1.12) | 0.96 (0.83–1.10) | 0.90 (0.74–1.09) | 0.88 (0.68–1.12) | 0.91 (0.78–1.07) | 0.87 (0.69–1.10) | 0.91 (0.67–1.21) |
| lag 0_5 | Male | 0.98 (0.86–1.11) | 0.90 (0.72–1.12) | 0.95 (0.82–1.10) | 0.88 (0.71–1.08) | 0.88 (0.67–1.15) | 0.91 (0.76–1.08) | 0.86 (0.67–1.09) | 0.90 (0.65–1.23) |
| lag 0_6 | Male | 0.99 (0.86–1.13) | 0.88 (0.70–1.12) | 0.96 (0.82–1.13) | 0.87 (0.70–1.09) | 0.88 (0.66–1.17) | 0.92 (0.77–1.11) | 0.87 (0.67–1.12) | 0.90 (0.64–1.26) |
| lag 0_7 | Male | 1.02 (0.87–1.19) | 0.88 (0.67–1.15) | 1.00 (0.83–1.20) | 0.88 (0.68–1.13) | 0.88 (0.64–1.22) | 0.96 (0.78–1.19) | 0.91 (0.68–1.20) | 0.91 (0.62–1.34) |
| lag 0 | Female | 1.01 (0.97–1.06) | 1.02 (0.93–1.11) | 1.00 (0.95–1.06) | 1.03 (0.95–1.11) | 1.02 (0.91–1.14) | 1.00 (0.93–1.07) | 1.01 (0.92–1.12) | 1.04 (0.91–1.19) |
| lag 0_1 | Female | 1.02 (0.96–1.10) | 1.04 (0.91–1.19) | 1.01 (0.93–1.10) | 1.06 (0.93–1.20) | 1.05 (0.89–1.23) | 1.01 (0.91–1.12) | 1.04 (0.89–1.21) | 1.08 (0.89–1.32) |
| lag 0_2 | Female | 1.04 (0.96–1.13) | 1.07 (0.92–1.24) | 1.03 (0.93–1.14) | 1.08 (0.94–1.25) | 1.08 (0.90–1.30) | 1.03 (0.91–1.16) | 1.07 (0.90–1.27) | 1.14 (0.91–1.41) |
| lag 0_3 | Female | 1.05 (0.96–1.15) | 1.09 (0.93–1.29) | 1.05 (0.94–1.17) | 1.11 (0.95–1.29) | 1.12 (0.92–1.36) | 1.05 (0.92–1.19) | 1.11 (0.93–1.33) | 1.19 (0.95–1.49) |
| lag 0_4 | Female | 1.06 (0.96–1.17) | 1.12 (0.94–1.34) | 1.06 (0.94–1.20) | 1.12 (0.95–1.33) | 1.15 (0.93–1.42) | 1.07 (0.93–1.23) | 1.14 (0.94–1.39) | 1.24 (0.97–1.59) |
| lag 0_5 | Female | 1.06 (0.95–1.18) | 1.14 (0.94–1.38) | 1.06 (0.93–1.21) | 1.13 (0.94–1.36) | 1.18 (0.94–1.48) | 1.08 (0.93–1.25) | 1.16 (0.94–1.42) | 1.28 (0.98–1.67) |
| lag 0_6 | Female | 1.05 (0.93–1.18) | 1.14 (0.93–1.40) | 1.05 (0.91–1.21) | 1.13 (0.93–1.37) | 1.19 (0.93–1.51) | 1.07 (0.91–1.26) | 1.15 (0.92–1.43) | 1.29 (0.97–1.71) |
| lag 0_7 | Female | 1.02 (0.89–1.17) | 1.13 (0.90–1.43) | 1.01 (0.86–1.19) | 1.11 (0.89–1.38) | 1.18 (0.89–1.56) | 1.05 (0.87–1.26) | 1.11 (0.87–1.42) | 1.28 (0.92–1.78) |
| lag 0 | First Nations | 1.02 (0.97–1.06) | 1.02 (0.94–1.11) | 1.01 (0.96–1.07) | 1.04 (0.96–1.13) | 1.04 (0.93–1.15) | 1.02 (0.95–1.09) | 1.06 (0.96–1.17) | 1.06 (0.93–1.21) |
| lag 0_1 | First Nations | 1.03 (0.96–1.10) | 1.04 (0.91–1.18) | 1.02 (0.94–1.11) | 1.07 (0.95–1.21) | 1.06 (0.90–1.24) | 1.03 (0.93–1.14) | 1.09 (0.94–1.27) | 1.09 (0.89–1.34) |
| lag 0_2 | First Nations | 1.04 (0.96–1.12) | 1.06 (0.91–1.23) | 1.03 (0.93–1.13) | 1.08 (0.94–1.24) | 1.07 (0.89–1.28) | 1.02 (0.91–1.15) | 1.09 (0.93–1.29) | 1.09 (0.88–1.36) |
| lag 0_3 | First Nations | 1.04 (0.95–1.14) | 1.07 (0.91–1.25) | 1.03 (0.93–1.15) | 1.07 (0.93–1.25) | 1.06 (0.87–1.28) | 1.01 (0.89–1.14) | 1.07 (0.90–1.28) | 1.07 (0.85–1.35) |
| lag 0_4 | First Nations | 1.04 (0.94–1.15) | 1.07 (0.90–1.28) | 1.03 (0.91–1.16) | 1.06 (0.90–1.25) | 1.04 (0.84–1.29) | 1.00 (0.87–1.14) | 1.04 (0.86–1.26) | 1.04 (0.81–1.34) |
| lag 0_5 | First Nations | 1.03 (0.92–1.14) | 1.05 (0.87–1.28) | 1.01 (0.89–1.15) | 1.04 (0.87–1.24) | 1.02 (0.81–1.28) | 0.98 (0.85–1.14) | 1.01 (0.82–1.24) | 1.01 (0.76–1.33) |
| lag 0_6 | First Nations | 1.00 (0.89–1.13) | 1.01 (0.83–1.25) | 0.99 (0.86–1.14) | 1.01 (0.83–1.22) | 0.99 (0.77–1.26) | 0.98 (0.83–1.15) | 0.99 (0.80–1.23) | 0.98 (0.73–1.32) |
| lag 0_7 | First Nations | 0.97 (0.84–1.11) | 0.96 (0.76–1.21) | 0.96 (0.82–1.13) | 0.98 (0.79–1.21) | 0.95 (0.72–1.26) | 0.98 (0.82–1.18) | 0.98 (0.77–1.25) | 0.96 (0.69–1.36) |
| lag 0 | Non-Indigenous | 1.02 (0.97–1.07) | 0.98 (0.88–1.09) | 0.99 (0.93–1.06) | 0.98 (0.89–1.08) | 0.93 (0.81–1.06) | 0.95 (0.87–1.03) | 0.93 (0.83–1.05) | 0.95 (0.81–1.11) |
| lag 0_1 | Non-Indigenous | 1.02 (0.94–1.10) | 0.97 (0.82–1.13) | 0.99 (0.89–1.09) | 0.96 (0.83–1.12) | 0.89 (0.73–1.09) | 0.93 (0.82–1.05) | 0.90 (0.75–1.08) | 0.95 (0.75–1.20) |
| lag 0_2 | Non-Indigenous | 1.01 (0.92–1.11) | 0.95 (0.80–1.14) | 0.98 (0.87–1.10) | 0.95 (0.81–1.13) | 0.89 (0.72–1.11) | 0.93 (0.81–1.07) | 0.90 (0.74–1.11) | 0.98 (0.76–1.27) |
| lag 0_3 | Non-Indigenous | 1.00 (0.90–1.11) | 0.94 (0.78–1.14) | 0.97 (0.86–1.11) | 0.95 (0.79–1.14) | 0.92 (0.73–1.16) | 0.95 (0.82–1.11) | 0.93 (0.75–1.15) | 1.04 (0.80–1.35) |
| lag 0_4 | Non-Indigenous | 1.00 (0.89–1.12) | 0.94 (0.77–1.16) | 0.98 (0.85–1.12) | 0.95 (0.78–1.15) | 0.97 (0.76–1.24) | 0.98 (0.83–1.15) | 0.96 (0.76–1.21) | 1.12 (0.84–1.48) |
| lag 0_5 | Non-Indigenous | 1.00 (0.88–1.14) | 0.96 (0.77–1.21) | 0.99 (0.84–1.15) | 0.96 (0.77–1.18) | 1.02 (0.78–1.33) | 1.01 (0.84–1.20) | 1.00 (0.78–1.28) | 1.18 (0.87–1.60) |
| lag 0_6 | Non-Indigenous | 1.03 (0.89–1.18) | 1.00 (0.79–1.27) | 1.01 (0.85–1.19) | 0.97 (0.78–1.22) | 1.08 (0.81–1.42) | 1.02 (0.85–1.23) | 1.02 (0.79–1.32) | 1.23 (0.89–1.69) |
| lag 0_7 | Non-Indigenous | 1.08 (0.92–1.26) | 1.06 (0.81–1.40) | 1.05 (0.86–1.27) | 1.00 (0.77–1.30) | 1.12 (0.81–1.55) | 1.02 (0.83–1.27) | 1.03 (0.77–1.38) | 1.24 (0.85–1.79) |
| lag 0 | <18 years | 0.99 (0.94–1.05) | 0.98 (0.88–1.10) | 0.97 (0.91–1.04) | 1.04 (0.94–1.16) | 1.01 (0.87–1.16) | 0.96 (0.88–1.04) | 1.03 (0.91–1.17) | 1.04 (0.88–1.24) |
| lag 0_1 | <18 years | 0.99 (0.90–1.07) | 0.96 (0.81–1.14) | 0.95 (0.85–1.06) | 1.06 (0.91–1.24) | 1.00 (0.81–1.23) | 0.92 (0.80–1.05) | 1.05 (0.87–1.27) | 1.06 (0.82–1.37) |
| lag 0_2 | <18 years | 0.98 (0.88–1.08) | 0.95 (0.78–1.15) | 0.92 (0.81–1.05) | 1.06 (0.88–1.27) | 0.98 (0.78–1.24) | 0.88 (0.75–1.03) | 1.06 (0.85–1.31) | 1.05 (0.80–1.39) |
| lag 0_3 | <18 years | 0.97 (0.86–1.08) | 0.93 (0.76–1.15) | 0.90 (0.79–1.04) | 1.04 (0.86–1.26) | 0.96 (0.75–1.23) | 0.85 (0.72–1.01) | 1.05 (0.84–1.32) | 1.03 (0.77–1.38) |
| lag 0_4 | <18 years | 0.96 (0.84–1.09) | 0.93 (0.74–1.16) | 0.89 (0.76–1.04) | 1.02 (0.83–1.26) | 0.95 (0.72–1.25) | 0.83 (0.70–1.00) | 1.05 (0.82–1.34) | 1.02 (0.74–1.40) |
| lag 0_5 | <18 years | 0.94 (0.82–1.09) | 0.93 (0.73–1.19) | 0.88 (0.74–1.04) | 1.02 (0.81–1.28) | 0.96 (0.71–1.29) | 0.83 (0.68–1.01) | 1.05 (0.81–1.37) | 1.03 (0.73–1.45) |
| lag 0_6 | <18 years | 0.93 (0.80–1.09) | 0.94 (0.73–1.23) | 0.87 (0.72–1.05) | 1.03 (0.81–1.31) | 1.00 (0.73–1.36) | 0.84 (0.68–1.04) | 1.06 (0.81–1.39) | 1.07 (0.74–1.53) |
| lag 0_7 | <18 years | 0.92 (0.77–1.09) | 0.97 (0.72–1.30) | 0.87 (0.70–1.07) | 1.07 (0.81–1.41) | 1.07 (0.75–1.52) | 0.88 (0.69–1.11) | 1.08 (0.80–1.46) | 1.15 (0.76–1.74) |
| lag 0 | 18-45 years | 1.03 (0.98–1.09) | 0.98 (0.89–1.09) | 1.03 (0.97–1.10) | 0.98 (0.89–1.08) | 0.93 (0.81–1.07) | 1.02 (0.94–1.10) | 1.00 (0.88–1.13) | 0.99 (0.84–1.17) |
| lag 0_1 | 18-45 years | 1.05 (0.97–1.14) | 0.98 (0.84–1.15) | 1.05 (0.95–1.16) | 0.96 (0.83–1.12) | 0.91 (0.74–1.12) | 1.03 (0.91–1.16) | 0.98 (0.82–1.18) | 1.00 (0.78–1.28) |
| lag 0_2 | 18-45 years | 1.05 (0.96–1.16) | 0.99 (0.82–1.19) | 1.05 (0.94–1.18) | 0.95 (0.80–1.13) | 0.93 (0.74–1.17) | 1.03 (0.89–1.19) | 0.96 (0.78–1.19) | 1.02 (0.78–1.35) |
| lag 0_3 | 18-45 years | 1.04 (0.94–1.16) | 1.00 (0.82–1.22) | 1.04 (0.92–1.19) | 0.94 (0.78–1.13) | 0.97 (0.76–1.23) | 1.03 (0.88–1.20) | 0.93 (0.75–1.17) | 1.05 (0.79–1.40) |
| lag 0_4 | 18-45 years | 1.03 (0.91–1.16) | 1.00 (0.80–1.24) | 1.03 (0.89–1.19) | 0.92 (0.75–1.13) | 0.99 (0.76–1.29) | 1.02 (0.86–1.21) | 0.91 (0.71–1.16) | 1.06 (0.78–1.46) |
| lag 0_5 | 18-45 years | 1.02 (0.89–1.16) | 0.97 (0.77–1.23) | 1.01 (0.87–1.19) | 0.89 (0.71–1.12) | 0.98 (0.73–1.31) | 1.01 (0.85–1.21) | 0.88 (0.67–1.14) | 1.04 (0.74–1.46) |
| lag 0_6 | 18-45 years | 1.01 (0.87–1.16) | 0.92 (0.71–1.19) | 1.00 (0.84–1.19) | 0.85 (0.67–1.08) | 0.91 (0.67–1.24) | 1.01 (0.83–1.22) | 0.85 (0.65–1.12) | 0.97 (0.67–1.39) |
| lag 0_7 | 18-45 years | 1.00 (0.85–1.18) | 0.84 (0.63–1.13) | 0.99 (0.81–1.20) | 0.80 (0.61–1.05) | 0.79 (0.55–1.12) | 1.00 (0.80–1.24) | 0.83 (0.61–1.13) | 0.86 (0.56–1.31) |
| lag 0 | >45 years | 1.03 (0.96–1.10) | 1.07 (0.94–1.22) | 1.02 (0.94–1.11) | 1.05 (0.93–1.19) | 1.04 (0.89–1.22) | 1.02 (0.92–1.12) | 1.00 (0.86–1.16) | 1.02 (0.84–1.24) |
| lag 0_1 | >45 years | 1.05 (0.95–1.17) | 1.13 (0.93–1.37) | 1.06 (0.93–1.20) | 1.10 (0.91–1.32) | 1.08 (0.85–1.37) | 1.05 (0.90–1.23) | 1.01 (0.81–1.27) | 1.05 (0.79–1.40) |
| lag 0_2 | >45 years | 1.08 (0.95–1.22) | 1.16 (0.93–1.45) | 1.10 (0.94–1.28) | 1.13 (0.92–1.39) | 1.11 (0.85–1.45) | 1.10 (0.92–1.32) | 1.04 (0.81–1.35) | 1.09 (0.80–1.49) |
| lag 0_3 | >45 years | 1.10 (0.96–1.26) | 1.19 (0.94–1.50) | 1.14 (0.97–1.35) | 1.16 (0.92–1.45) | 1.14 (0.86–1.50) | 1.16 (0.96–1.41) | 1.09 (0.83–1.42) | 1.14 (0.83–1.58) |
| lag 0_4 | >45 years | 1.13 (0.96–1.31) | 1.22 (0.94–1.57) | 1.18 (0.99–1.42) | 1.18 (0.92–1.51) | 1.18 (0.87–1.59) | 1.22 (0.99–1.50) | 1.14 (0.85–1.52) | 1.21 (0.85–1.72) |
| lag 0_5 | >45 years | 1.15 (0.97–1.37) | 1.25 (0.95–1.66) | 1.22 (1.00–1.49) | 1.21 (0.92–1.58) | 1.23 (0.89–1.71) | 1.25 (1.00–1.57) | 1.18 (0.87–1.61) | 1.28 (0.88–1.88) |
| lag 0_6 | >45 years | 1.18 (0.98–1.42) | 1.31 (0.97–1.77) | 1.24 (1.00–1.54) | 1.23 (0.93–1.64) | 1.31 (0.93–1.86) | 1.26 (0.99–1.60) | 1.22 (0.88–1.67) | 1.37 (0.92–2.04) |
| lag 0_7 | >45 years | 1.21 (0.98–1.49) | 1.40 (1.00–1.96) | 1.25 (0.97–1.60) | 1.27 (0.92–1.75) | 1.43 (0.96–2.13) | 1.22 (0.93–1.61) | 1.24 (0.86–1.77) | 1.46 (0.92–2.32) |

**Supplementary Table 4** Sensitivity analyses showing cumulative relative risk of all cause ED visits at lag 0-2 days for heatwaves

|  | **cb df 4** | **cb df 5** | **Excluding humidity** | **Humidity df 4** | **Humidity df 5** | **NS time df 8** | **NS time df 9** |
| --- | --- | --- | --- | --- | --- | --- | --- |
|  | RR (95%CI) | RR (95%CI) | RR (95%CI) | RR (95%CI) | RR (95%CI) | RR (95%CI) | RR (95%CI) |
| **HW_1** | 1.03 (0.96–1.10) | 1.03 (0.96–1.10) | 1.03 (0.97–1.10) | 1.03 (0.97–1.10) | 1.03 (0.97–1.10) | 1.01 (0.94–1.07) | 1.02 (0.95-1.08) |
| **HW_2** | 1.02 (0.93–1.13) | 1.02 (0.92–1.13) | 1.02 (0.93–1.13) | 1.03 (0.93–1.13) | 1.03 (0.93–1.13) | 0.99 (0.90–1.08) | 1.00 (0.91-1.10) |
| **HW_3** | 1.03 (0.91–1.17) | 1.02 (0.90–1.16) | 1.03 (0.91–1.17) | 1.02 (0.90–1.15) | 1.02 (0.90–1.15) | 0.98 (0.87–1.11) | 0.99 (0.88-1.12) |
| **HW_4** | 1.01 (0.94–1.10) | 1.01 (0.93–1.10) | 1.01 (0.93–1.09) | 1.02 (0.94–1.10) | 1.02 (0.95–1.10) | 0.99 (0.91–1.07) | 1.00 (0.92-1.08) |
| **HW_5** | 1.03 (0.92–1.16) | 1.03 (0.91–1.16) | 1.04 (0.92–1.16) | 1.03 (0.92–1.16) | 1.03 (0.93–1.15) | 0.99 (0.88–1.11) | 1.01 (0.90-1.13) |
| **HW_6** | 1.01 (0.87–1.18) | 1.03 (0.88–1.20) | 1.01 (0.87–1.18) | 1.00 (0.86–1.16) | 1.00 (0.87–1.15) | 0.96 (0.83–1.11) | 0.96 (0.83-1.11) |
| **HW_7** | 0.99 (0.90–1.09) | 0.97 (0.88–1.08) | 0.97 (0.87–1.08) | 0.99 (0.90–1.10) | 1.00 (0.91–1.09) | 0.96 (0.88–1.06) | 0.97 (0.88-1.07) |
| **HW_8** | 1.02 (0.88–1.17) | 1.02 (0.88–1.18) | 1.02 (0.87–1.19) | 1.02 (0.89–1.17) | 1.02 (0.89–1.16) | 0.96 (0.84–1.10) | 0.98 (0.86-1.13) |
| **HW_9** | 1.05 (0.88–1.26) | 1.03 (0.87–1.22) | 1.06 (0.88–1.27) | 1.04 (0.89–1.21) | 1.04 (0.89–1.21) | 1.00 (0.85–1.17) | 1.00 (0.84-1.20) |
